# Development of Cannabidiol-Loaded PLGA Microspheres for Long-Acting Injectable Delivery: Evaluation of Poly(2-ethyl-2-oxazoline) as an Alternative to Poly(ethylene glycol)

**DOI:** 10.3390/pharmaceutics18030336

**Published:** 2026-03-08

**Authors:** Thabata Muta, Haripriya Koppisetti, Sanjay Garg

**Affiliations:** Centre for Pharmaceutical Innovation (CPI), School of Pharmacy and Biomedical Science, Adelaide University, Adelaide, SA 5000, Australiaharipriya.koppisetti@adelaide.edu.au (H.K.)

**Keywords:** cannabidiol, long-acting injectable, PLGA microspheres, poly(2-ethyl-2-oxazoline), poly(ethylene glycol), sustained drug release, in vitro release testing, biodegradable polymers

## Abstract

**Background/Objectives**: Current clinical evidence suggests that cannabidiol (CBD) demonstrates therapeutic potential in the management of chronic pain, particularly in conditions involving inflammation. However, its therapeutic potential is severely limited by poor oral bioavailability, extensive first-pass metabolism, and the need for frequent high-dose administration, which compromises patient adherence and tolerability. Long-acting injectable (LAI) delivery systems offer a strategy to overcome these limitations by providing sustained plasma concentrations and reducing dosing frequency. This study aimed to develop and optimise CBD-loaded poly (lactic-co-glycolic acid) (PLGA) microspheres for LAI delivery and to evaluate poly(2-ethyl-2-oxazoline) (POx) as a functional and biocompatible alternative to the conventionally used poly (ethylene glycol) (PEG). **Methods**: CBD-loaded microspheres were prepared using emulsion–solvent evaporation technique. The formulations were optimised based on entrapment efficiency (EE), drug loading (DL), particle size distribution, surface morphology, thermal behaviour, in vitro release kinetics, and cytocompatibility using NIH 3T3 fibroblasts. Multiple in vitro release methodologies, including dialysis bag, shaking-flask, and USP Apparatus IV, were evaluated to identify the most discriminative and practical approach for long-term release assessment. **Results**: The optimised POx-based microspheres demonstrated superior control over particle size, yielding significantly smaller and more uniform particles compared with PEG-based microspheres (124 ± 1.47 µm vs. 218 ± 13.5 µm, respectively). Differential scanning calorimetry (DSC) confirmed molecular dispersion of CBD within the polymer matrix. In vitro release studies demonstrated sustained drug release over 20 days. **Conclusions**: POx represents a promising alternative to PEG for the formulation of CBD-loaded PLGA microspheres, offering enhanced physicochemical stability and biological compatibility. This platform supports the development of safe and effective long-acting injectable CBD therapies and consideration of POx as an alternative to PEG.

## 1. Introduction

Cannabidiol (CBD), the primary non-intoxicating phyto cannabinoid derived from *Cannabis sativa*, has gained significant clinical traction for the management of chronic refractory conditions, including Dravet syndrome, neuropathic pain, and anxiety disorders [1]. Unlike conventional opioids, which present high risks of dependence and respiratory depression, or traditional antiepileptics associated with severe cognitive impairment, CBD offers a favourable safety profile with no abuse potential [2]. However, the therapeutic utility of CBD is severely compromised by its hostile physicochemical properties. Being highly lipophilic (logP~6.3) and subject to extensive first-pass metabolism, CBD exhibits a low and poor oral bioavailability estimated at less than 10% in humans [3]. Consequently, patients are often required to administer high daily doses to achieve therapeutic plasma levels, leading to gastrointestinal adverse effects and poor adherence [4].

To overcome the pharmacokinetic limitations of oral cannabidiol, research has increasingly focused on parenteral long-acting injectable (LAI) strategies [5,6,7], including liposomes [8], nanocrystalline suspensions [9], and in situ forming implants (ISFIs) [10]. However, many of these systems fail to achieve the therapeutic profile required for chronic CBD administration. Simple oil-based depots and nanocrystalline suspensions typically function as sustained boluses rather than true depots, exhibiting rapid onset (T_max_ approximately 40 min to 3 h) and insufficient duration for monthly dosing [9,11]. While liposomal carriers can achieve sustained release, they have been associated with severe local tissue reactions, such as fibrinopurulent cellulitis, upon subcutaneous administration [8]. Similarly, although ISFIs offer prolonged drug release, they are often hindered by formulation challenges, including high viscosity that complicates injectability and susceptibility to uncontrolled initial burst release driven by solvent efflux [10]. Consequently, biodegradable polymeric microspheres have emerged as a superior platform, offering tuneable erosion kinetics that ensure consistent, multi-week release with minimal burst effects and superior physicochemical stability compared to lipid-based or solvent-dependent systems [11].

PLGA stands as a gold standard in long-acting injections primarily due to its intrinsic biodegradability, where it hydrolyses into lactic and glycolic acids that are safely eliminated via the Krebs cycle as CO_2_ and water [12]. This safety profile is reinforced by its extensive biocompatibility and minimal inflammatory response, which coupled with a long history of FDA approved products like Lupron Depot, significantly minimising the risks in regulatory pathway for new formulations [13]. Furthermore, its exceptional tuneability allows researchers to precisely engineer release kinetics from weeks to months by adjusting the molecular weight and lactide:glycolide ratio [14]. Also, its solubility in common organic solvents, including dimethyl sulfoxide and ethyl acetate, facilitates robust manufacturing processes, such as solvent evaporation and phase separation, which are essential for consistent microsphere fabrication [15]. Also, hydrophilic additives are often incorporated into the organic phase to modulate porosity and hydration rates. Poly(ethylene glycol) (PEG) has traditionally been the polymer of choice for this purpose due to its established history of us [14]. However, in the recent times, the status of PEG has been challenging. Accumulating evidence suggests that PEG is susceptible to oxidative degradation and can elicit an immune response in the form of anti-PEG antibodies, leading to the “Accelerated Blood Clearance” (ABC) phenomenon upon repeated administration [16,17,18].

These limitations highlight the need to explore alternative hydrophilic polymers with improved stability and biocompatibility. Poly(2-ethyl-2-oxazoline) (POx) has emerged as a leading candidate to replace PEG [19], due to its structural flexibility, excellent biocompatibility, and “stealth” properties similar to PEG, yet it possesses a lower viscosity and higher stability against oxidative stress [20,21]. The exploration of POx as a versatile pharmaceutical excipient has expanded significantly across various drug delivery architectures. To date, POx-based formulations have been successfully utilised in the development of high-capacity micelles for taxoids and other poorly water-soluble drugs, where the polymer’s side-chain flexibility facilitates superior drug loading and stability compared to traditional PEG-based systems [22]. Furthermore, POx has demonstrated efficacy as a “stealth” coating for lipid nanoparticles (LNPs) and liposomes, offering a robust alternative that avoids the immunogenic responses, such as the Accelerated Blood Clearance (ABC) phenomenon, associated with PEGylated carriers [19]. In the field of polymer–drug conjugates and hydrogels, POx’s tunable thermal responsiveness and low viscosity have been leveraged to create injectable depots for protein and small molecule delivery [23]. Despite these advancements in liquid, micellar, and nanoparticle systems, the use of POx as a functional matrix modifier within solid PLGA microspheres, specifically for the delivery of highly lipophilic cannabinoids like CBD, remains largely unexplored [21]. This study aims to fill that gap by evaluating how the unique amphiphilic architecture of POx influences the stabilisation and release kinetics of solid polymeric depots.

Therefore, the primary objective of this study is to investigate the potential of POx as an alternative to PEG in the formulations of CBD-loaded microspheres. This study provides a comparative analysis of these POx and PEG, examining their influence on microsphere morphology, entrapment efficiency (EE), drug loading (DL) and sustained release profiles, as well as a critical assessment of in vitro release testing methods for injectable microspheres. The rationale behind this strategy was to exploit the amphiphilic nature of POx to enhance drug–polymer binding, potentially improving drug encapsulation and promoting a more controlled release profile.

## 2. Materials and Methods

### 2.1. Materials

Cannabidiol (CBD) crystals in powder form were kindly provided by Green Dispensary Compounding (Adelaide, Australia). High Performance Liquid Chromatography (HPLC)-grade methanol and acetonitrile (ACN) were obtained from EMD Millipore^®^ (Billerica, MA, USA). Dimethyl sulfoxide (DMSO), dichloromethane (DCM), and ethyl acetate (EA) were procured from Chemsupply Australia. Poly (Lactic-co-glycolic) acid (MW 85:15, 72:25) (PLGA) was sourced from Nomisma (Vadodara, India). Poly(2-ethyl-2-oxazoline) (POx) and polyvinyl alcohol (PVA) from Sigma Aldrich (Sydney, Australia). Polyethylene glycol (400) (PEG) was procured from Glentham Life Sciences. A Sartorius ultra-pure water system was utilised in all studies (Goettingen, Lower Saxony, Germany). Dialysis tubing cellulose membrane average flat width 25 mm (1.0 in.), Dulbecco’s Modified Eagle Medium (DMEM), Dulbecco’s Phosphate-Buffered Saline (DPBS), Foetal bovine serum (FBS), l-glutamine, penicillin/streptomycin (10,000 U/mL), trypan blue solution, and a 96-well clear flat-bottom plate were obtained from Merck Pty Ltd. (Sydney, Australia). Biorender was used to produce the graphs and figures (accessed on 20 January 2026).

The following Table 1 provides the physicochemical properties, safety profile, and key specifications of Poly(2-ethyl-2-oxazoline) (POx), detailing its structural formula, appearance, solubility in various solvents, and biocompatibility. These characteristics were integral to evaluating POx as a promising polymer in the development of microspheres [24].

### 2.2. Preparation of CBD-Loaded Microspheres

CBD-loaded microspheres were prepared using an oil-in-water (O/W) emulsion–solvent evaporation method. The accurate quantities of CBD, PLGA, and the hydrophilic additives (POx or PEG) were weighed and transferred into 15 mL Falcon tubes. The components (100 mg of CBD, 500 mg of PLGA and 125 mg of POx) were dissolved in 10 mL of the selected organic solvent (EA) and vortexed to ensure complete dissolution. The organic phase was added dropwise into 30 mL of 0.5% *w*/*v* aqueous polyvinyl alcohol (PVA) solution preheated to 40 °C and kept under constant magnetic stirring at 500 rpm for 1 h. Stirring was maintained at room temperature to stabilise the initial dispersion for another 1 h. Further, the primary emulsion formed was added dropwise into 400 mL of 0.5% *w*/*v* PVA solution at room temperature. Solvent evaporation and particle hardening were achieved by magnetic stirring at ambient temperature for 6–8 h. The resulting hardened microparticles were collected by centrifugation (3700 rpm for 15 min) and washed three times with ultra-pure water to remove residual PVA and non-encapsulated drug. The collected microspheres were immediately frozen at −80 °C and lyophilised for 24 h to obtain a free-flowing powder, which was stored at −20 °C [25,26,27].

### 2.3. Preliminary Screening

To identify the critical material attributes (CMAs) and critical process parameters (CPPs) influencing the critical quality attributes (CQAs) of the LAI, a systematic preliminary screening was performed, as summarised in Table 2. This screening evaluated the effects of CPPs on the CQAs, such as (i) drug loading (DL), and (ii) in vitro drug release profile. The CMAs investigated include (a) polymeric matrix composition, where PLGA of different molecular weights, 85:15 or 75:25, were evaluated at different ratios ranging from 10 to 40% *w*/*w*, (b) organic solvents, where DCM and EA were evaluated due to the high solubility of CBD in these media (>250 mg/mL in DCM and >500 mg/mL in EA) [5,15], and (c) hydrophilic polymeric additives such as POx were evaluated from 0.25 to 10% *w*/*v*. POx concentrations were further narrowed to 0.5 to 5% *w*/*v* for accurate evaluation. As established in previous studies for CBD microspheres [5], to reduce the droplet size and form a pre-colloidal suspension, the coarse primary emulsion was processed using an ultrasonic probe (Qsonica Q125 Sonicator, Newtown, CT, USA) and maintained on ice at 4 °C to prevent thermal degradation. The effect of CPPs—such as sonication power, sonication on-off time, and total sonication time—was assessed. All the screening factors are described in Table 2. For this systematic screening phase, data are reported as single values to identify qualitative formulation trends. For all optimised lead formulations and final characterisation studies, measurements were performed in triplicate (*n* = 3) and results are expressed as mean ± standard deviation (SD) to ensure reproducibility.

### 2.4. Analytical Method

In order to quantify the CBD loaded into the microspheres and its in vitro drug release, HPLC analysis was performed under the conditions previously established and optimised by Abdella et al. [28]. An isocratic separation was achieved using a Luna C8 analytical column (5 µm, 100 A°, 250 × 4.6 mm) maintained at 30 °C. The column was integrated into the Shimadzu HPLC system (Shimadzu Corporation, Kyoto, Japan) equipped with a photodiode array detector (LC-20ADXR), degasser (DGU-20A3), system controller (CBM-20A), autosampler (SIL-20AHT), pump (LC-20AD), and LC solution chromopac data processor. The mobile phase consisted of acetonitrile and Milli-Q water (80:20% *w*/*v*) with a flow rate of 1.0 mL/min. A 20 µL sample was injected, and detection was carried out at 220 nm, where CBD exhibited a retention time of 7.3 min [29].

### 2.5. Drug Loading

Accurately weighed 10 ± 0.5 mg of microspheres were dissolved in 1 mL of DCM and 9 mL of mobile phase (80:20, ACN: ultra-pure water, % *v*/*v*), and vortexed for 1 h in a multi-tube vortex mixer (Model MTV1, Ratek, Melbourne, Australia) to completely extract the drug from the polymeric matrix. Following vortex, the samples were subjected to centrifugation at 3700 rpm for 10 min, and the supernatants were collected and quantified using HPLC. Measured in triplicate, the percentage DL and EE of the microspheres were calculated using the following Equations (1) and (2) [7].

(1) $$Drug\;loading=\frac{Amount\;of\;CBD\;encapsulated}{Total\;mass\;of\;microspheres}\times 100$$

(2) $$Encapsulation\;Efficiency=\frac{Amount\;of\;CBD\;encapsulated}{Total\;amount\;of\;CBD\;added}\times 100$$

### 2.6. In Vitro Release Study

All formulations were subjected to in vitro drug release studies using a shake-flask method in phosphate-buffered saline (PBS, pH 7.4). Briefly, accurately weighed microspheres (10.0 ± 0.5 mg) were suspended in 10 mL of PBS (pH 7.4) containing 0.5% (*w*/*v*) Tween 20 to maintain sink conditions. The suspensions were incubated under constant agitation at 37 ± 0.5 °C with a shaking speed of 50 rpm. At predetermined time points (days 1, 3, 7, 10, 15, 20, 25, and 30), the entire flask and release medium was collected, and a 3 mL aliquot was withdrawn, filtered through a 0.45 µm cellulose acetate syringe filter (Sartorius, Göttingen, Germany), and analysed for CBD content using HPLC [29]. To evaluate the robustness and relevance of the selected in vitro release methodology, the shake-flask approach was compared with release studies performed using a dialysis bag method [30], and a USP Apparatus IV (flow-through cell) method [31]. All studies were performed in triplicate.

#### 2.6.1. Dialysis Bag Diffusion Technique

Release studies were conducted using the dialysis bag diffusion method, a technique widely employed for lipophilic compounds microparticles to ensure physical separation of the carrier from the release medium while maintaining sink conditions [30]. Accurately weighed microspheres (10 mg) were suspended in 1 mL of release medium and transferred into a cellulose ester dialysis membrane (MWCO: 12–14 kDa; Spectrum Laboratories). The sealed bag was immersed in a vial containing 9 mL of phosphate-buffered saline (PBS, pH 7.4) with 0.5% (*w*/*v*) Tween 20 to enhance CBD solubility. The system incubated in an orbital shaker (Ratek Instruments Pty Ltd., RSM7DC, Victoria, Australia) at 37 °C with an agitation speed of 200 rpm. At predetermined time points, the entire volume of the release media was withdrawn and replaced with fresh, pre-warmed medium.

#### 2.6.2. Shake-Flask Method

Accurately weighed 10 mg of microspheres were added to 10 mL of release media composed of PBS (pH 7.4) with 0.5% (*w*/*v*) Tween 20 and incubated at 37 °C in an orbital shaker with agitation speed of 200 rpm. At each sampling point, the entire release media was withdrawn and filtered through a 0.45 µm PVDF syringe filter. This step was implemented to ensure that only the solubilised drug was quantified and to prevent the removal of undissolved microspheres. Following sampling, the equal amounts of media was replaced with fresh buffer to maintain constant volume [30].

#### 2.6.3. USP Apparatus IV (Flow-Through Cell)

The USP Apparatus IV (flow-through cell) system (Sotax CE 7smart, Sotax AG, Allschwil, Switzerland) is considered as the standard for biorelevant testing of parenteral formulations due to its ability to mimic the hydrodynamics of the subcutaneous and intramuscular routes of administration, which involves exposure to continuous small volumes of physiological fluids [32]. To further correlate the in vitro release methodologies mentioned in Section 2.6.1 and Section 2.6.2, the microspheres were subjected to release study using USP IV. Accurately weighed 10 mg of microspheres were mixed with glass beads (1 mm diameter) to prevent particle aggregation and ensure laminar flow. This mixture was loaded into a standard 12.5 mm flow-through cell. A glass microfiber filter and a ruby bead were placed at the cell inlet and outlet to retain the microspheres. The system was operated in a closed-loop configuration with a flow rate of 1.5 mL/min at 37 ± 0.5 °C. At scheduled time intervals, samples were automatically withdrawn from the reservoir, filtered, and analysed.

### 2.7. Particle Size Distribution

The particle size distribution of the microspheres was determined using a Mastersizer 3000 (Malvern Panalytical, Malvern, UK). Particle size analysis was performed using a wet dispersion technique using a Hydro EV/LV unit filled with approximately 400 mL of reverse osmosis (RO) water as the dispersant. For the measurement, 10–20 mg of the microspheres in lyophilised powder form were added directly into the dispersion tank under moderate stirring (approx. 2000 rpm) to ensure a homogeneous suspension and prevent sedimentation. The sample amount was adjusted to achieve a laser obscuration range of 5–15%, ensuring an optimal signal-to-noise ratio while minimising multiple scattering. Particle size was calculated using the Mie theory, with a refractive index of 1.59 assigned to the polymer and 1.33 to the aqueous dispersant. Results were expressed as volume-weighted percentiles (D_10_, D_50_, D_90_), and the Span value calculated as (D_90_/D_10_/D_50_).

### 2.8. Surface Morphology

A Zeiss Merlin FE-SEM (Oberkochen, Germany) was used to evaluate the morphological characteristics of the CBD-loaded microspheres. The pure drug (CBD), and the formulations having POx (M1), PEG (M4), and additive-free (M7) were freely spread all over the double-sided conductive carbon tape placed on aluminium stubs. To further enhance the surface conductivity, the samples were coated with a thin layer of platinum using Agar high resolution sputter coater (Model: Platinum Target, Rotherham, UK). Imaging was performed under high vacuum conditions at an accelerating voltage of 1–2 kV, depending on sample sensitivity, at a magnification range of 50–2000×.

### 2.9. Differential Scanning Calorimetry (DSC)

DSC was performed using a DSC250 (TA Instruments, New Castle, DE, USA) to evaluate the onset temperature, melting point, width of melting events (WME), enthalpy, and crystallinity index (CI) of the pure drug, microspheres and the physical mixture with the same ratio between drug and polymers. Approximately 3 mg of each sample was sealed in an aluminium pan and analysed from 25 to 200 °C at a ramp rate of 10 °C/min. Nitrogen was used as a purge gas at a flow rate of 50 mL/min.

### 2.10. Cell Viability

The biocompatibility of the microspheres was evaluated by assessing their effects on fibroblast viability and metabolic activity using NIH 3T3 cells and a standard MTT assay. NIH 3T3 fibroblasts are widely used as a reference cell line for cytocompatibility studies due to their prevalence in connective tissues and their well-established responsiveness to biomaterial exposure [33]. Cytotoxicity testing was performed in accordance with ISO 10993-5 guidelines [34]. NIH 3T3 cells were cultured in DMEM supplemented with 10% Foetal bovine serum (FBS) and 1% penicillin–streptomycin and maintained at 37 °C with 5% CO_2_.

For the assay, 1 × 10^4^ cells per well were seeded into 96-well plates and allowed to adhere overnight. Release media obtained from microsphere formulations M1, M4, and M7 were sterilised using 0.2 µm filters (Sartorius, Göttingen, Germany), and subsequently diluted in DMEM to yield final CBD concentrations of 0, 0.01, 0.1, and 1 µg/mL, as quantified previously by HPLC [29]. The diluted samples were added to the wells and incubated for 24 h. Following the treatment, the media was replaced with 0.5% (*w*/*v*) MTT solution, and plates were incubated for 3 h at 37 °C. The supernatant was then removed, and the resulting formazan crystals were dissolved in DMSO. Absorbance was measured at 540 nm using a microplate reader (PerkinElmer, Waltham, MA, USA). Cell viability was expressed as a percentage relative to untreated controls.

## 3. Results

### 3.1. Preliminary Screening and Optimisation

The preliminary screening was performed to assess the impact of various formulation parameters on particle formation and DL (Table 3). Based on effect of CMAs on CPPs and CQAs in the screening, the formulations F5, F6, and F19 were identified to be the lead compositions for further optimisation based to achieve a tailored in vitro drug release over 20 days. This formulation intentionally targets an initial burst release of 10% within the first 24 h to provide a rapid symptomatic relief for pain or anxiety, while the subsequent sustained release facilitated by the PLGA matrix ensures prolonged therapeutic level [27,35]. The incorporation of PEG or POx plays a key role in modulating matrix porosity and water uptake, thereby enabling a more controlled initial burst release profile compared with conventional PLGA-only systems [12,36]. The formulations were designed to achieve a minimum DL of 20–30% [5].

**Figure 1:**
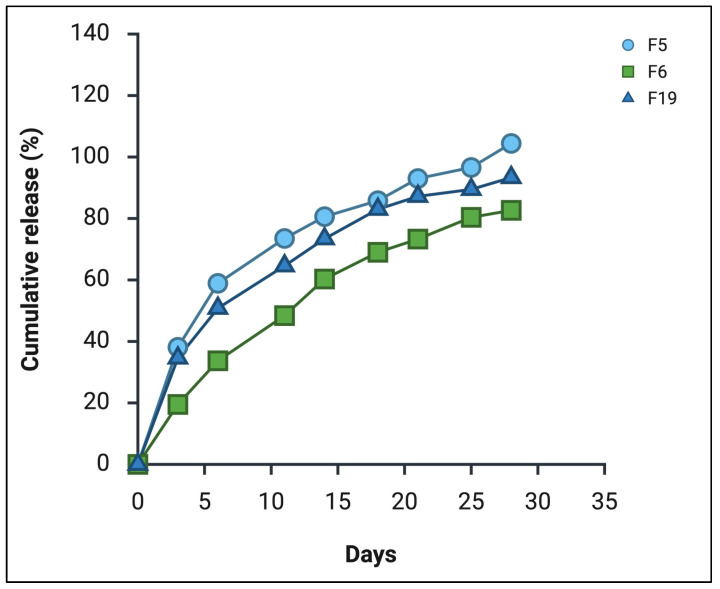
In vitro release profile of CBD from selected preliminary formulations (F5, F6, and F19) over 28 days (*n* = 1).

Previous studies on CBD-PLGA microparticles have demonstrated the feasibility of high-loading strategies, with some formulations achieving loadings as high as 50% [27]. Furthermore, given the high lipophilicity of CBD, which favours its retention within the hydrophobic. Based on the %DL and in vitro drug release profile, the formulations F5, F6, and F19 were identified to be the optimum with %DL > 40% and sustained in vitro drug release with 100% (F5), 82.6% (F6), and 93.3% (F19) over a period of 28 days (Figure 1).

Following preliminary screening, selected formulations (F5, F6, and F19) were subsequently prepared using POx, PEG, or without additives (M1–M9) to enable a direct comparison of POx and PEG and to evaluate their influence on EE, DL, particle size, and in vitro release behaviour. The compositions of these formulations are summarised in Table 4. However, during scale-up for more in-depth comparative evaluation, it was observed that sonication induced significant particle agglomeration. This effect is likely attributable to interparticle collision energies exceeding repulsive forces, thereby promoting droplet coalescence and subsequent agglomeration [37,38].

To address this limitation, in further optimisation trails, sonication was excluded and preparation method was refined to a more scalable and reproducible method as mentioned in Section 2.2. Observing the agglomeration when preparing the preliminary formulation selected, besides sonication, it was noticed that this might have been caused also due to the slow solvent evaporation. The optimised and scalable method resulted in a highly concentrated primary emulsion increasing the viscosity of the dispersed phase, providing steric stabilisation of droplets and suppressing Brownian motion, thereby minimising coalescence. Subsequent addition into a secondary PVA solution promoted rapid interfacial solvent diffusion and particle hardening, effectively “locking in” particle morphology before agglomeration could occur [38]. During the secondary optimisation phase (Table 5), the concentrations of CBD and PLGA were maintained constant across formulations, and higher concentrations of PLGA copolymer ratios (75:25 and 85:15) were evaluated to improve the %DL. The concentrations of POx or PEG in organic solvent, % *w*/*v* PVA in aqueous phase, and the choice of organic solvent were kept identical to those used during the initial preliminary screening (Table 3) to minimise confounding variables. In addition, maintaining consistent components concentration allowed for a direct comparison of the effects of PLGA copolymer composition (75:25 versus 85:15) on microsphere properties without introducing additional formulation-dependent variability.

**Figure 2:**
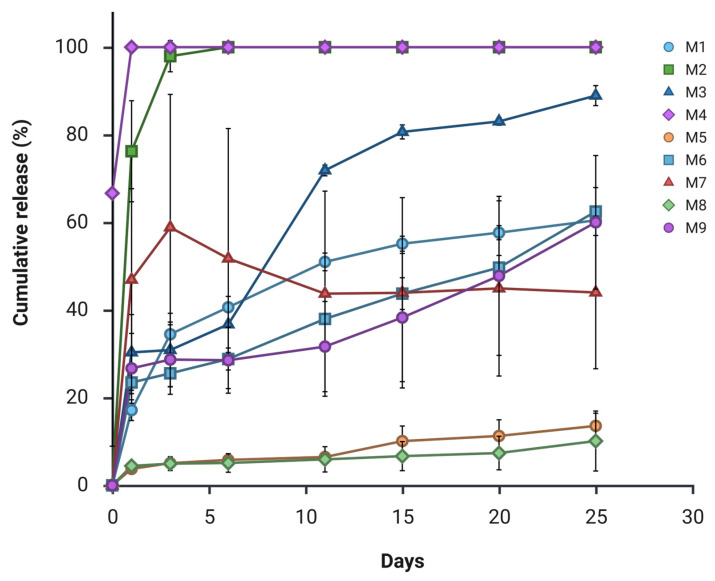
Cumulative CBD release profiles for POx, PEG, and additive-free formulations using the shaking flask method (*n* = 3).

To ensure the robustness and reproducibility of the observed trends, formulations M1 to M9 were subjected to in vitro release studies performed in triplicate. As shown in Figure 2, formulation M2 exhibited an extremely rapid release profile, with 76.3 ± 9.4% of the encapsulated CBD released within the first few hours. A similarly rapid release behaviour was observed for formulation M4, indicating limited capacity for sustained drug delivery in both systems. Formulation M7 displayed inconsistent release behaviour, characterised by an initial burst at Day 1 and Day 3, followed by negligible additional release beyond Day 6.

In addition, formulations M3 and M9 also demonstrated an early burst release at Day 1, reaching 30.3 ± 7.0% and 26.7 ± 6.5% cumulative release, respectively. These values exceeded the predefined target for initial release and therefore rendered these formulations unsuitable for the intended long-acting injectable profile. In contrast, formulations M5 and M8 exhibited minimal drug release over the study period, suggesting excessive drug entrapment within a dense polymer matrix and limited polymer erosion [12]. Among all formulations evaluated, M1 and M6 emerged as the most promising candidates in this initial screening, demonstrating a controlled release kinetics characterised by a controlled initial release followed by sustained drug delivery over the intended timeframe.

### 3.2. Quantification of Drug

**Figure 3:**
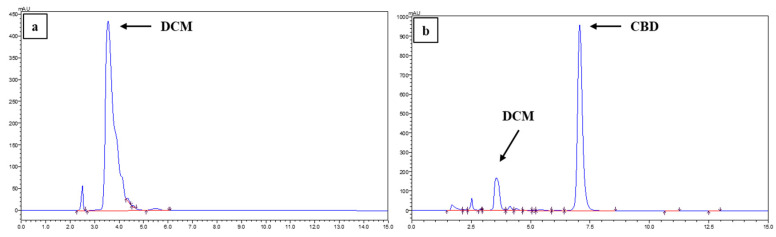
Representative HPLC chromatograms evaluating analytical specificity. (**a**) Injection of pure dichloromethane (DCM); (**b**) formulation M1 sample obtained from encapsulation efficiency (EE) analysis. The distinct retention times demonstrate clear separation between the residual solvent and the cannabidiol (CBD) peaks, confirming the absence of analytical interference.

To assess the potential for analytical interference arising from the co-elution of DCM and CBD, the chromatographic profiles were evaluated. As depicted in Figure 3, the DCM peak eluted at approximately 3.54 min, whereas the CBD peak was observed at 7.00 min. This distinct separation confirms the absence of peak overlap, thereby precluding any analytical interference from the solvent.

### 3.3. Drug Loading, Entrapment Efficiency and Particle Size Distribution

During the optimisation phase of the preliminary screening, formulations M1 to M9 show significant differences in particle size and DL that are strongly influenced by excipient selection (Table 6). The incorporation of PEG 400 (formulations M4–M6) generally resulted in higher EE (52.9–81.3%). However, this improvement was accompanied by a substantial increase in particle size. Formulations M3, M4, M6, and M8 exhibited mean particle diameters exceeding 100 µm, rendering them unsuitable for parenteral administration. Particles of this magnitude significantly impair syringeability and are associated with a heightened risk of needle occlusion and localised irritation at the injection site [39]. In contrast, additive-free formulations M7 and M9, produced significantly smaller particles (<18 µm). However, particles below 10 µm are typically associated with increased surface area and accelerated drug release [39]. Regarding particle size, formulations M1, M2, M5 and M7 falls within the generally accepted range for injectability (10–100 µm) [39]. Notably, M1 and M6 were the only formulations to demonstrate superior sustained release profiles (Figure 2). However, M6 exhibited a mean particle size exceeding the parenteral threshold and an extremely low drug loading (9.5%). Based on the synthesis of these characterisation data, M1 emerges as the optimal formulation for further development.

However, when addressing batch scalability and issues with particle agglomeration, the secondary optimisation phase also aimed to improve DL and EE, as well as to confirm the accuracy and reproducibility of particle size measurements obtained during the preliminary pre-formulation stage presented in Table 6. This optimisation led to the identification of a final composition (Table 5), in which the POx-based formulation (M1F) exhibited a markedly higher EE (93.7 ± 6.9%) compared with the PEG-based analogue (84.8 ± 7.8%), as shown in Table 7.

Unlike PEG, which can disrupt polymer–drug miscibility and influence phase behaviour and drug crystallinity in PLGA systems, POx such as poly(2-ethyl-2-oxazoline) exhibits amphiphilic behaviour that can promote more homogeneous drug dispersion within the polymer matrix [23]. Consistent with these findings, formulation M1F (Pox-based) once again demonstrated superior particle size control and batch uniformity compared with M4F (PEG-based), as evidenced by its smaller mean particle size and narrower size distribution (lower standard deviation) reported in Table 6.

### 3.4. Surface Morphology

**Figure 4:**
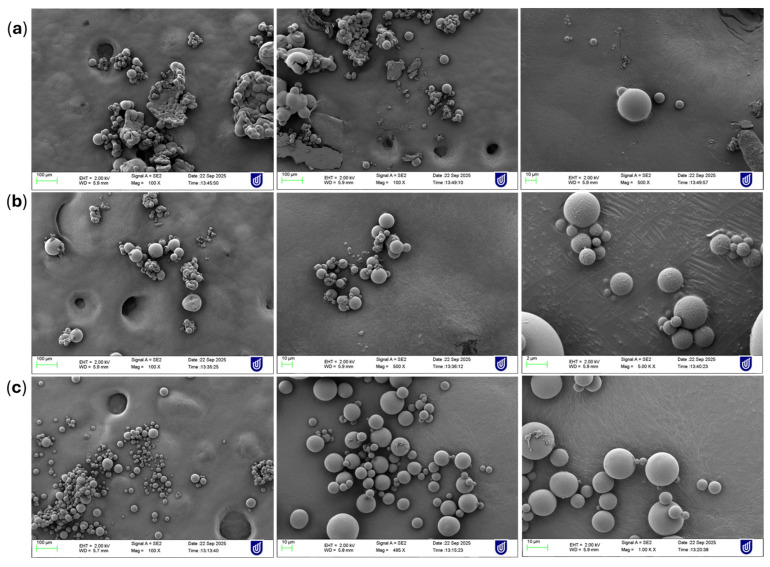
Images of microspheres showing surface morphology examined using SEM. (**a**) Formulation having no additive (M7F), (**b**) formulation having PEG (M4F), and (**c**) formulation having POx (M1F).

SEM analysis was conducted to compare the surface characteristics of microspheres prepared using PEG, POx and without any hydrophilic polymer. Differences in the morphology were observed across the three formulations, highlighting the influence of polymer additives on microsphere formulation. As shown in Figure 4, microspheres prepared without any hydrophilic polymer display a broader size distribution with surface irregularities and particle aggregation. This suggests a suboptimal droplet stabilisation during the emulsification process. The absence of a steric stabiliser likely contributed to inconsistent polymer precipitation and heterogeneous particle formation.

In contrast, M4F and M1F showed an improved spherical structure and smoother surfaces compared to M7F. However, minor particle aggregations and variability in particle size are still evident in PEG microspheres. This indicated that PEG enhanced the droplet stabilisation to some extent; its hydrophilic nature might have resulted in accelerated solvent diffusion, leading to localised surface collapse during solidification [40,41]. On the other hand, POx-based microspheres demonstrated the most desirable morphology among the three systems. The SEM images revealed uniformly sized, well-defined spherical particles with smooth and continuous surfaces with minimal structural defects. The enhanced uniformity suggests that POx provided superior steric stabilisation, promoting controlled solvent evaporation and consistent polymer hardening. This resulted in a narrow size distribution and highly reproducible microsphere architecture. Overall, the SEM evaluation confirms that the incorporation of POx significantly improves the microsphere morphology compared to PEG and non-aided formulations, producing particles that are more suitable for controlled release applications.

### 3.5. Differential Scanning Calorimetry (DSC)

**Figure 5:**
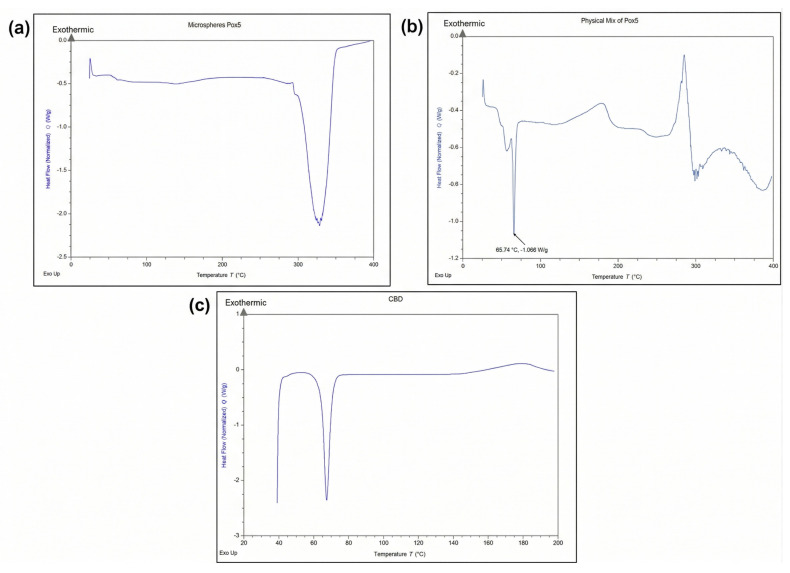
DSC thermograms of (**a**) CBD-loaded microspheres (M1F), (**b**) physical mixture, and (**c**) pure CBD.

DSC was performed to evaluate the thermal behaviour and physical state of the drug and polymer within the microsphere formulations. Samples of pure CBD, drug-loaded microspheres and a physical mixture of POx, PLGA and CBD were analysed to assess the potential interactions and confirm the successful encapsulation. The thermogram, as shown in Figure 5c, confirms the crystalline nature of CBD with a melting point at 66 °C. In contrast, this characteristic peak was absent or significantly reduced in microsphere formulations Figure 5a, indicating that the drug was molecularly dispersed and converted to an amorphous form within the polymer matrix. The polymer exhibits an initial thermal transition corresponding to glass transition temperature at 30–40 °C and degradation at 220 °C. In the physical mixture Figure 5b, the CBD peak was retained and showed a melting peak at 66 °C, indicating no interactions in the physical mixture.

### 3.6. In Vitro Release Studies

In the preliminary study (Figure 1), the formulation F5 exhibited a complete drug release (100%) by Day 25, whereas the optimised final formulation M1F achieved comparable complete release by Day 20, confirming the reproducibility and robustness of the release behaviour and optimised method of preparation (Section 2.2). It is important to note that the release methodologies employed in these two phases were not identical. The preliminary screening utilised a conventional “in-sink” sampling approach, which maintains constant sink conditions but may inadvertently exclude microspheres during media replacement, potentially biassing the apparent release rate. In contrast, the final study analysed the entire release flask at each time point to ensure that intact microspheres were not inadvertently removed with the supernatant, thereby providing a more comprehensive assessment of cumulative release. Differences in sampling approach and sink maintenance can substantially influence apparent release kinetics, particularly for long-acting formulations where polymer hydration, particle swelling, and diffusional barriers evolve over extended durations [1,2].

**Figure 6:**
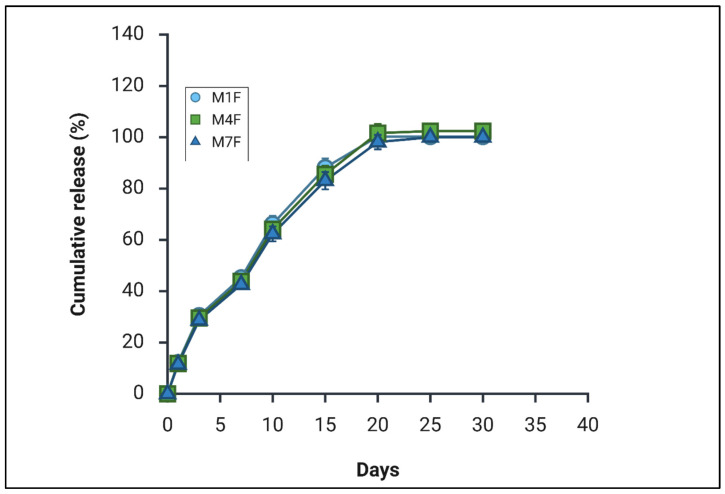
Release profiles of formulations M1F, M4F, and M7F using the total-release shaking flask method (sink conditions maintained via total volume collection), *n* = 3.

When comparing the final release profiles of formulations M1F, M4F, and M7F (Figure 6), a high degree of similarity was observed across all three systems. This finding contrasts with the preliminary screening results (Figure 2), where POx-based formulations, prepared in small scale and using sonication demonstrated superior release performance compared with their PEG-based counterparts. In the optimised formulations, the release profiles of POx- and PEG-containing systems converged, suggesting that process optimisation and methodological rigour exert a greater influence on release kinetics than surfactant identity alone under the final preparation and analytical conditions.

### 3.7. Comparison of Different Studies

To further assess the robustness and relevance of the release behaviour, formulation M1 was evaluated using multiple in vitro release methodologies. The translation of LAI microspheres from bench to bedside is frequently hindered by the absence of standardised regulatory guidelines for in vitro release testing [42]. Unlike oral dosage forms, for which compendial dissolution methods are well established, no single in vitro method has been universally approved as predictive of the in vivo performance of parenteral systems. This often results in poor in vitro–in vivo correlation (IVIVC) and limited clinical translatability of laboratory data [32]. Consequently, the development and validation of a robust and discriminative in vitro release method represent a critical step in LAI formulation development.

This challenge is particularly pronounced for formulations intended to provide extended drug release over prolonged durations (>21 days) [42]. Conventional release methods frequently fail to predict in vivo behaviour due to factors such as saturation of the release media, instability of small molecule drugs, and difficulties in maintaining sink condition, especially for highly lipophilic compounds such as cannabidiol [30]. To identify the most discriminative and biorelevant release method for the developed CBD-loaded microspheres, three established in vitro release setups were systematically evaluated, the dialysis bag diffusion technique, shaking flask method and USP Apparatus IV (flow-through cell). All three methodologies have been previously employed for the assessment of extended-release parenteral dosage forms [32,43,44].

**Figure 7:**
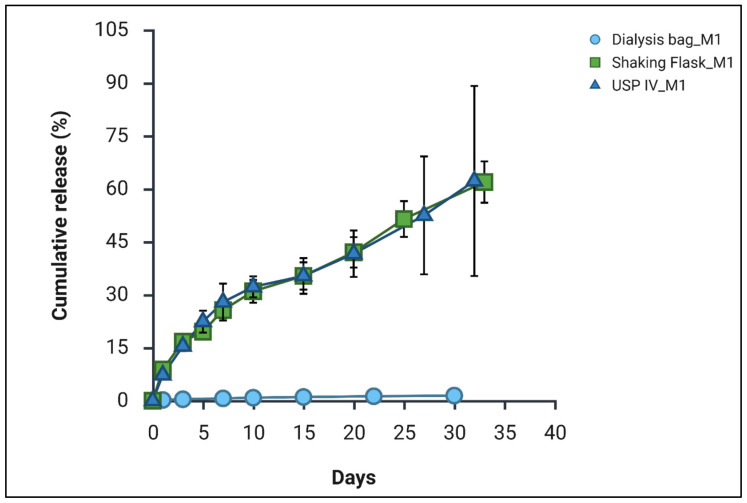
Comparison of different drug release methods. Standard deviation (SD) for the dialysis bag method was <0.08% (*n* = 3).

The comparative release profiles obtained for M1 are represented in Figure 7. The results clearly demonstrate that the dialysis bag method is unsuitable for the assessment of the free pure drug and CBD release from PLGA-based microspheres, as evidenced by the consistently low recovery of the active pharmaceutical ingredient (API) in the release medium. This limitation persisted even after the addition of 1 mL of release medium inside the dialysis bag to facilitate polymer swelling and drug diffusion [45], suggesting that the dialysis membrane itself imposes a diffusional barrier [45], as also observed in previous studies performed without internal release medium. In contrast, both the USP Apparatus IV and the shaking flask method produced comparable release profiles. These findings are also in close agreement with data obtained using the previously employed release method (Figure 1).

Although USP Apparatus IV is widely regarded as a robust and biorelevant system for evaluating parenteral formulations and has been extensively applied for the in vitro release assessment of highly lipophilic compounds [42]. Similarly, from the literature, shake-flask method is most commonly used and recognised particularly for PLGA-based microspheres and have shown good in vitro–in vivo correlation offering experimental flexibility, scalability, and reliable maintenance of sink conditions [11,46]. This approach enables simultaneous evaluation of multiple samples making it particularly suitable for formulation optimisation and comparative release studies of hydrophobic drugs such as cannabidiol.

### 3.8. Cell Viability

**Figure 8:**
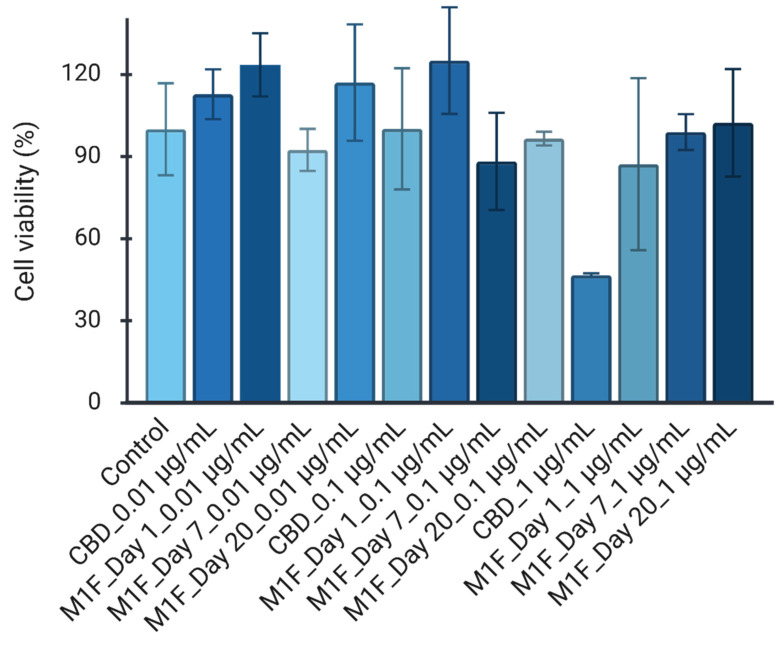
MTT assay for M1F (optimised POx microsphere) at Day 1, 7 and 20; *n* = 3.

The evaluation of cellular compatibility is a prerequisite for the development of parenteral formulations, as the local accumulation of lipophilic APIs or polymeric by-products can induce tissue necrosis and inflammation at the injection site [47]. To assess the safety profile of the novel POx based microspheres (M1), cell viability was quantified using the MTT assay. The administration of non-encapsulated CBD elicited a marked dose-dependent reduction in cellular viability. While lower concentrations (0.01–0.1 µg/mL) were well tolerated, exposure to a concentration of 1 µg/mL resulted in a precipitous decline in cell survival to 46.6 ± 0.5% (Figure 8). This finding aligns with established literature demonstrating that, at micromolar concentrations, cannabinoids can trigger apoptosis in mammalian cells via the induction of oxidative stress and the activation of caspase pathways [48,49].

Encapsulation of CBD within the polymeric matrix, regardless of the additive used, significantly mitigated the cytotoxic effects observed with the free drug. At an equivalent drug loading of 1 µg/mL, maintained cell viability above the levels seen with free CBD. This “shielding effect” confirms that the PLGA matrix effectively sequesters the drug, releasing it at a controlled rate that avoids saturating the cellular microenvironment [25]. M1F displayed a stable and physiological viability profile (Day 1: 87.2%; Day 7: 98.9%; Day 20: 102.3%) with no significant deviation from the untreated control group (Figure 8). M1F formulation exhibited a larger mean diameter (~89 µm) and amphiphilic surface properties. This indicates that POx polymer formed stable micelles within the release media, effectively solubilising the hydrophobic CBD. This micellar encapsulation acts as a ‘protective shield,’ preventing the direct membrane disruption typically caused by free CBD and without inducing metabolic stress, thereby maintaining high cell viability despite the identical drug load [22].

## 4. Discussion and Conclusions

This study demonstrates that CBD-loaded PLGA microspheres prepared using an emulsion–solvent evaporation strategy and incorporating poly(2-ethyl-2-oxazoline) (POx) shows a comparable in vitro release behaviour and uniform particle size distribution to PEG-modified and additive-free formulations when manufactured in a small scale. However, despite these advantages, in the preliminary screening the POx formulations exhibited suboptimal entrapment efficiency (EE of 32.98%) and yielded insufficient batch quantities for comprehensive physicochemical and biological characterisation. These limitations necessitated the transition of a highly concentrated O/W emulsion into a secondary PVA solution phase to enable scalable production and improved formulation particle size and EE.

Optimisation of the process resulted in a final formulation (M1F) in which sustained CBD release was successfully achieved. While no significant differences in cumulative release profiles were observed between POx-, PEG-, and additive-free systems under the optimised preparation method, marked differences were evident in critical quality attributes. These variations persist despite the high concentration of PVA used across all formulations, which serves as the primary extrinsic stabiliser to prevent droplet coalescence and ensure initial particle formation [12]. While PVA governs the external physical stability of the emulsion, the inclusion of POx or PEG as intrinsic matrix modifiers appears to dictate the internal architecture and drug–polymer miscibility [38]. Unlike PVA, which is largely washed away during the triple-wash centrifugation step (Section 2.2), POx and PEG are incorporated within the organic phase (Section 2.3). This means they are integrated into the PLGA matrix itself. The “shielding effect” and improved molecular dispersion observed in the POx-based M1F formulation (Section 3.8) suggest that POx influences the internal microstructure and drug–polymer interactions [40]. If PVA were the only determinant of properties, the additive-free (M7F), PEG (M4F), and POx (M1F) formulations would behave identically. However, SEM results (Figure 4) and Table 7 show that even with consistent PVA concentrations, the optimised POx formulation (M1F) exhibited significantly improved particle size control (124 ± 1.47 µm), alongside enhanced EE (93.7 ± 6.9%) and DL (19.8 ± 2.0%), outperforming both PEG-based (M4F) and additive-free counterparts (M7F). DLS analysis confirmed that CBD was molecularly dispersed and converted to an amorphous form within the polymer matrix and SEM confirmed the superior performance of the POx-based system, which, unlike the irregular and aggregated additive-free or PEG formulations, produced uniformly defined, spherical microspheres with smooth surfaces and minimal structural defects. These findings suggest that although PVA is indeed a powerful stabiliser essential for preventing droplet coalescence, it primarily governs the external morphology and physical stability of the emulsion. The hydrophilic additives (POx/PEG) function as matrix modifiers that dictate the internal drug distribution, entrapment efficiency, and long-term release kinetics—attributes that PVA alone cannot modulate [15,23].

The mechanistic superiority of POx over PEG in this system is likely governed by the distinct chemical architecture and interfacial behaviour of the poly(2-oxazoline) chains. While PEG is a linear polyether that provides simple hydrophilicity, POx (specifically poly(2-ethyl-2-oxazoline)) possesses a tertiary amide group in its repeating unit, granting it an amphiphilic character that enhances dipole–dipole interactions with the lipophilic CBD molecules [22,23]. This chemical affinity promotes a more homogeneous molecular dispersion of the drug within the PLGA matrix, directly contributing to the significantly higher EE observed in POx-based formulations (M1F) compared to PEG-based (M4F) or additive-free (M7F) controls. Furthermore, the lower organic phase viscosity of POx, combined with its superior oxidative stability, facilitates more uniform solvent diffusion kinetics during the evaporation phase [40,41]. Unlike PEG, which is prone to oxidative stress and can cause localised surface collapse during particle hardening, POx provides robust steric stabilisation that ‘locks in’ a uniform, spherical morphology with minimal structural defects, as co[40,41nfirmed by SEM imaging. This optimised internal architecture not only ensures particle uniformity but also prevents the premature drug crystallisation often observed with simpler hydrophilic modifiers [18,21].

Cell viability studies further emphasise the importance of controlled drug release and surface chemistry. Free CBD induced pronounced cytotoxicity at the highest concentration tested, consistent with concentration-dependent cannabinoid-mediated oxidative stress and apoptotic signalling. In contrast, POx-based microspheres exhibited a stable and physiologically relevant cytocompatibility profile. Collectively, these findings identify POx-modified PLGA microspheres as a robust and biocompatible platform for long-acting injectable delivery of CBD.

## 5. Future Research Directions

Moving forward, the focus should shift toward expanding the POx-based platform from a specific CBD formulation into a broader, more versatile delivery system. A particularly promising direction is the development of composite “microsphere-in-gel” systems. By embedding CBD-Pox-loaded microspheres within an in situ forming, thermo-responsive POx hydrogel, we can create a “double-barrier” architecture. This setup should help minimise initial burst release and prevent particles from migrating away from the injection site, potentially extending the therapeutic window from weeks to several months. The refinement of POx chemical architecture will be central to this progress. By systematically adjusting molecular weights and polydispersity, we can effectively “tune” the matrix density and chain entanglement. This provides a precise way to control drug–polymer interactions and erosion kinetics, allowing for release profiles that are specifically tailored to the lipophilic nature of CBD or other poorly water-soluble drugs.

For future considerations for the clinical use, in vivo pharmacokinetic and histopathological studies are to be established to understand the in vitro-in vivo correlation and to confirm the platform’s long-term safety. A key priority will be evaluating local tissue responses to validate POx as a non-immunogenic alternative to PEG, specifically to determine if it can avoid the Accelerated Blood Clearance (ABC) phenomenon often seen with PEGylated systems. Finally, investigating amphiphilic POx block copolymers could significantly improve drug loading capacity. By creating hydrophobic “pockets” within the microsphere, we can stabilise CBD in its amorphous state, preventing crystallisation. Testing this strategy with a wider range of lipophilic drugs will help establish POx as a foundational, clinically relevant excipient for the next generation of long-acting injectables.

## Figures and Tables

**Table 1:** Physicochemical properties, safety profile, and specifications of Poly(2-ethyl-2-oxazoline) (5–7 cSt).

Linear Formula	[-N(COC2H5)CH2CH2-]n
PubChem Substance ID	329755635
Appearance (Colour) *	White to yellow to light orange
Appearance (Form) *	Chips or crystals
Viscosity (5–7 cSt) *	Low dynamic viscosity in solution
Solubility	High in water, Ethanol, Chloroform, and ethyl acetate
Safety and Toxicity	Non-toxic, non-immunogenic, and biocompatible
Availability	Commercially available (e.g., Sigma-Aldrich, Ultroxa^®^)

* Values for viscosity and appearance are based on the manufacturer’s specifications for Product #372846. Solubility was confirmed experimentally in both aqueous and organic phases (EA/DCM) during the formulation process. Non-immunogenicity and “stealth” properties are supported by the absence of the “Accelerated Blood Clearance” (ABC) phenomenon in the literature comparisons with PEG-based systems.

**Table 2:** Evaluated ranges for CMAs, CPPs and CQAs during preliminary formulation screening and optimisation.

Factor Category	Variable	Low-Level (−1)	Mid-Level (0)	High-Level (+1)
CMAs	PLGA Ratio (Lactide:Glycolide)	75:25	-	85:15
	PLGA Concentration (% *w*/*v*)	10	25	40
	CBD Concentration (% *w*/*v*)	5	12.5	20
	POx (% *w*/*v*)	2.5	6.25	10
	Organic Solvent	DCM	-	EA
	External Phase PVA (%)	0.5	2.75	5
CPPs	Sonication Power (%)	20	45	70
	Sonication Total Time (s)	30	75	120
CQAs	Drug Loading (DL) (%)	Target: >20%		
	Initial Burst Release (Day 3)	Target: ≈30%		
	Cumulative Release (28 days)	Target: Sustained		

**Table 3:** Preliminary screening and optimisation of composition.

Formulation Runs	Critical Material Attributes (CMAs)						Critical Process Parameters (CPPs)				Critical Quality Attributes (CQAs)	
	CBD in Solution (% *w*/*v*)	PLGA (% *w*/*v*)	POx (% *w*/*v*)	PVA (% *w*/*v*)	PLGA MW	Solvent	Sonication Power (%)	Sonication on-Time (s)	Sonication off Time (s)	Sonication Total Time (s)	Drug Loading (%)	In Vitro Release Profile (Day 3)
1	5	10	10	0.5	85:15	DCM	20	3	30	75	32.3	Initial BR of 49%
2	20	10	10	5	85:15	EA	70	3	17.5	30	68.3	Initial BR 63%
3	5	40	10	5	85:15	DCM	70	9	5	120	13.2	Very slow release
4	5	10	2.5	5	85:15	EA	20	3	5	120	31.8	Very slow release
5	20	10	2.5	0.5	75:25	EA	20	9	30	30	56.7	Initial BR 38% followed by SR
6	12.5	10	10	5	75:25	DCM	20	15	5	30	44.0	Initial BR 19% followed by SR
7	20	10	2.5	5	85:15	DCM	45	15	30	120	78.9	Very rapid drug release
8	5	40	2.5	0.5	75:25	DCM	20	15	17.5	120	13.6	Very slow release
9	5	10	10	2.75	75:25	EA	70	15	30	120	>100	Very rapid drug release
10	20	40	2.5	5	75:25	EA	70	15	5	75	43.5	Initial BR 46%
11	12.5	25	6.25	2.75	75:25	DCM	45	9	17.5	75	37.1	Initial BR 19% followed by SR
12	20	10	6.25	0.5	75:25	DCM	70	3	5	120	14.4	Very slow release
13	20	25	10	0.5	85:15	EA	20	15	5	120	59.1	Very slow release
14	12.5	25	6.25	2.75	85:15	EA	45	9	17.5	75	48.1	Slow SR release
15	12.5	40	2.5	0.5	85:15	EA	70	3	30	120	30.9	Very slow release
16	5	40	10	0.5	75:25	EA	45	3	5	30	12.6	Very slow release
17	5	10	2.5	0.5	85:15	DCM	70	15	5	30	32.8	Slow SR release
18	20	40	2.5	2.75	85:15	DCM	20	3	5	30	39.5	Initial BR 18% followed by SR
19	5	40	6.25	5	85:15	EA	20	15	30	30	49.2	Initial BR 34% followed by SR
20	5	25	2.5	5	75:25	DCM	70	3	30	30	35.9	Very slow release
21	20	40	10	0.5	85:15	DCM	70	15	30	30	38.7	Slow SR release
22	20	40	10	5	75:25	EA	20	3	30	120	37.1	Initial BR 31% followed by very slow release

**Table 4:** Composition of preliminary formulations comparing POx, PEG, and additive-free controls.

Parameters	Formulation	CBD (% *w*/*v*)	PEG (% *w*/*v*)	POx (% *w*/*v*)	PLGA 75:25 (% *w*/*v*)	PLGA 85:15 (% *w*/*v*)	PVA (% *w*/*v*)	Solvent (mL)	
								EA	DCM
Preliminary screening run 5	M1	20	-	2.5	10	-	0.5	10	-
	M4		2.5	-		-			-
	M7		-	-		-			-
Preliminary screening run 6	M2	12.5	-	10	10	-	5	-	
	M5		10	-		-		-	10
	M8		-	-		-		-	
Preliminary screening run 19	M3	5	-	6.25	-	40	5		-
	M6		6.25	-	-			10	-
	M9		-	-	-				-

**Table 5:** Final composition of optimised formulations: effects of batch scale-up, sonication removal, and secondary PVA (0.5%) solution addition on agglomeration prevention.

Parameters	Formulation	CBD (% *w*/*v*)	PEG (% *w*/*v*)	POx (% *w*/*v*)	PLGA 75:25 (% *w*/*v*)	PLGA 85:15 (% *w*/*v*)	PVA (% *w*/*v*)	Solvent (mL)	
								EA	DCM
Preliminary screening run 5	M1F	10	-	2.5	50	-	0.5		-
	M4F	10	2.5	-	50	-	0.5	10	-
	M7F	10	-	-	50	-	5		-
Preliminary screening run 6	M2F	10	-	10	50	-	5	-	
	M5F	10	10	-	50	-	5	-	10
	M8F	10	-	-	50	-	5	-	
Preliminary screening run 19	M3F	10	-	6.25	-	50	5		-
	M6F	10	6.25	-	-	50	5	10	-
	M9F	10	-	-	-	50	5		-

**Table 6:** Drug loading, entrapment efficiency, and particle size distribution of POx, PEG, and additive-free formulations during secondary screening.

Formulation	EE (%)	DL (%)	Particle Size Avg D_90_ (μm)
M1	33.0 ± 5.4	22.4 ± 2.6	89.8 ± 1.5
M2	42.4 ± 1.4	19.7 ± 1.2	44.9 ± 1.3
M3	47.1 ± 7.1	5.3 ± 1.9	234.8 ± 1.7
M4	52.9 ± 5.8	38.0 ± 1.7	859.7 ± 1.8
M5	81.3 ± 7.9	47.3 ± 3.3	98.1 ± 1.8
M6	76.8 ± 1.7	9.5 ± 1.5	337.6 ± 1.7
M7	85.6 ± 1.2	30.1 ± 2.7	17.3 ± 1.2
M8	78.0 ± 3.8	37.0 ± 5.3	1176.7 ± 0.9
M9	5.8 ± 1.5	5.7 ± 1.8	10.7 ± 1.4

**Table 7:** Comparison of encapsulation efficiency, drug loading, and particle size for final POx, PEG, and additive-free formulations.

Samples	EE %	DL %	Particle Size			
			Mean	1 × Std Dev	1RSD (%)	Span
			Dx (90) (μm)			
M1F	93.7 ± 6.9	19.8 ± 2.0	124	1.47	1.19	1.576
M4F	84.8 ± 7.8	22.8 ± 2.1	218	13.5	6.22	2.379
M7F	87.3 ± 1.4	25.2 ± 6.7	-	-	-	-

## Data Availability

The original contributions presented in this study are included in the article/Supplementary Materials. Further inquiries can be directed to the corresponding author.

## Supplementary Materials

The following supporting information can be downloaded at: https://www.mdpi.com/article/10.3390/pharmaceutics18030336/s1, Figure S1: Formulation Particle Size Analysis—Comparing formulations’ (M1F, M2F, M4F, and M5F) particle size distribution using the Mastersizer; Figure S2: Additional SEM micrographs at various magnifications.

## Abbreviations

The following abbreviations are used in this manuscript:

CBD	Cannabidiol
LAI	Long-acting injectable
PLGA	Poly (lactic-co-glycolic acid)
POx	Poly(2-ethyl-2-oxazoline)
PEG	Poly (ethylene glycol)
EE	Entrapment efficiency
DL	Drug loading
DSC	Differential scanning calorimetry
ISFIs	In situ forming implants
ABC	Accelerated blood clearance
ACN	Acetonitrile
DMSO	Dimethyl sulfoxide
DCM	Dichloromethane
EA	Ethyl acetate
PVA	Polyvinyl alcohol
DMEM	Dulbecco’s modified eagle medium
DPBS	Dulbecco’s phosphate-buffered saline
FBS	Foetal bovine serum
CMAs	Critical material attributes
CPPs	Critical process parameters
CQAs	Critical quality attributes
PBS	Phosphate-buffered saline
CI	Crystallinity index
WME	Width of melting events
HPLC	High Performance Liquid Chromatography
SEM	Scanning Electron Microscopy
IVIVC	In vitro–in vivo correlation
API	Active pharmaceutical ingredient

## References

[1] Devinsky O., Cilio M.R., Cross H., Fernandez-Ruiz J., French J., Hill C., Katz R., Di Marzo V., Jutras-Aswad D., Notcutt W.G. (2014). Cannabidiol: Pharmacology and potential therapeutic role in epilepsy and other neuropsychiatric disorders. Epilepsia.

[2] Louis R., World Health Organization (2018). CANNABIDIOL (CBD) Critical Review Report Expert Committee on Drug Dependence Fortieth Meeting.

[3] Millar S.A., Stone N.L., Yates A.S., O’SUllivan S.E. (2018). A Systematic Review on the Pharmacokinetics of Cannabidiol in Humans. Front. Pharmacol..

[4] Taylor L., Gidal B., Blakey G., Tayo B., Morrison G. (2018). A Phase I, Randomized, Double-Blind, Placebo-Controlled, Single Ascending Dose, Multiple Dose, and Food Effect Trial of the Safety, Tolerability and Pharmacokinetics of Highly Purified Cannabidiol in Healthy Subjects. CNS Drugs.

[5] Fraguas-Sánchez A.I., Torres-Suárez A.I., Cohen M., Delie F., Bastida-Ruiz D., Yart L., Martin-Sabroso C., Fernández-Carballido A. (2020). PLGA Nanoparticles for the Intraperitoneal Administration of CBD in the Treatment of Ovarian Cancer: In Vitro and In Ovo Assessment. Pharmaceutics.

[6] Davachi S.M., Vazquez M., Soleimani M., Hajmohammadi Z., Mohajer M., Jameie S.B., Khanmohammadi M., Najafi R., Bagher Z., Hassanzadeh S. (2024). Effectiveness of the injectable hyaluronic acid-based microparticles loaded with cannabidiol on rat sciatic nerve injury model. Int. J. Biol. Macromol..

[7] Fraguas-Sánchez A., Fernández-Carballido A., Simancas-Herbada R., Martin-Sabroso C., Torres-Suárez A. (2020). CBD loaded microparticles as a potential formulation to improve paclitaxel and doxorubicin-based chemotherapy in breast cancer. Int. J. Pharm..

[8] Shilo-Benjamini Y., Cern A., Zilbersheid D., Hod A., Lavy E., Barasch D., Barenholz Y. (2022). A Case Report of Subcutaneously Injected Liposomal Cannabidiol Formulation Used as a Compassion Therapy for Pain Management in a Dog. Front. Veter-Sci..

[9] Fu X., Xu S., Li Z., Chen K., Fan H., Wang Y., Xie Z., Kou L., Zhang S. (2022). Enhanced Intramuscular Bioavailability of Cannabidiol Using Nanocrystals: Formulation, In Vitro Appraisal, and Pharmacokinetics. AAPS PharmSciTech.

[10] Lozza I., Martín-Sabroso C., Torres-Suárez A.I., Fraguas-Sánchez A.I. (2024). In situ forming PLA and PLGA implants for the parenteral administration of Cannabidiol. Int. J. Pharm..

[11] Fraguas-Sánchez A.I., Hernán D., Montejo C., Poklis J.L., Lichtman A.H., Torres-Suárez A.I. (2024). Polycaprolactone microparticles for the subcutaneous administration of cannabidiol: In vitro and in vivo release. Drug Deliv. Transl. Res..

[12] Makadia H.K., Siegel S.J. (2011). Poly Lactic-co-Glycolic Acid (PLGA) as Biodegradable Controlled Drug Delivery Carrier. Polymers.

[13] Anderson J.M., Shive M.S. (2012). Biodegradation and biocompatibility of PLA and PLGA microspheres. Adv. Drug Deliv. Rev..

[14] Fredenberg S., Wahlgren M., Reslow M., Axelsson A. (2011). The mechanisms of drug release in poly(lactic-co-glycolic acid)-based drug delivery systems—A review. Int. J. Pharm..

[15] Wischke C., Schwendeman S.P. (2008). Principles of encapsulating hydrophobic drugs in PLA/PLGA microparticles. Int. J. Pharm..

[16] Garay R.P., El-Gewely R., Armstrong J.K., Garratty G., Richette P. (2012). Antibodies against polyethylene glycol in healthy subjects and in patients treated with PEG-conjugated agents. Expert Opin. Drug Deliv..

[17] Knop K., Hoogenboom R., Fischer D., Schubert U.S. (2010). Poly(ethylene glycol) in drug delivery: Pros and cons as well as potential alternatives. Angew. Chem. Int. Ed..

[18] Berger M., Degey M., Curnel A., Evrard B., Piel G. (2025). The benefits of emerging alternatives to PEG for lipid nanoparticle RNA delivery systems. Nanomedicine.

[19] van Zyl D.G., Mendes L.P., Semper R.P., Rueckert C., Baumhof P. (2024). Poly(2-methyl-2-oxazoline) as a polyethylene glycol alternative for lipid nanoparticle formulation. Front. Drug Deliv..

[20] Huo R.-P., Zhang X., Huang X.-R., Li J.-L., Sun C.-C. (2011). Direct ab initio dynamics study of radical C_4_H (X^2^Σ^+^) + CH_4_ reaction. J. Phys. Chem. A.

[21] Sedlacek O., Richard H. (2019). Drug Delivery Systems Based on Poly(2-Oxazoline)s and Poly(2-Oxazine)s. Adv. Ther..

[22] He Z., Schulz A., Wan X., Seitz J., Bludau H., Alakhova D.Y., Darr D.B., Perou C.M., Jordan R., Ojima I. (2015). Poly(2-oxazoline) based micelles with high capacity for 3rd generation taxoids: Preparation, in vitro and in vivo evaluation. J. Control. Release.

[23] Luxenhofer R., Han Y., Schulz A., Tong J., He Z., Kabanov A.V., Jordan R. (2012). Poly(2-oxazoline)s as polymer therapeutics. Macromol. Rapid Commun..

[24] Sigma-Aldrich (2026). Poly(2-ethyl-2-oxazoline). Product Number 372846. https://www.sigmaaldrich.com/AU/en/product/aldrich/372846?srsltid=AfmBOor2MZGRtOvHtblxjDeOyMW-M02QmvPg7wN_eyheYg9QhpqP2LhU.

[25] de la Ossa H.P., Lorente M., Gil-Alegre M.E., Torres S., García-Taboada E., Del Rosario Aberturas M., Molpeceres J., Velasco G., Torres-Suárez A.I. (2013). Local delivery of cannabinoid-loaded microparticles inhibits tumor growth in a murine xenograft model of glioblastoma multiforme. PLoS ONE.

[26] Jin Z., Zhan Y., Zheng L., Wei Q., Xu S., Qin Z. (2023). Cannabidiol-Loaded Poly Lactic-Co-Glycolic Acid Nanoparticles with Improved Bioavailability as a Potential for Osteoarthritis Therapeutic. ChemBioChem.

[27] David C., de Souza J.F., Silva A.F., Grazioli G., Barboza A.S., Lund R.G., Fajardo A.R., Moraes R.R. (2024). Cannabidiol-loaded microparticles embedded in a porous hydrogel matrix for biomedical applications. J. Mater. Sci. Mater. Med..

[28] Abdella S., Kim S., Afinjuomo F., Song Y., Upton R., Garg S. (2023). Harnessing the Synergistic Potential of 3D Printed Buccal Films and Nanostructured Lipid Carriers (NLCs) For Personalised Cannabidiol Delivery. Drug Deliv. Transl. Res..

[29] Muta T., Khetan R., Song Y., Garg S. (2025). Optimising Cannabidiol Delivery: Improving Water Solubility and Permeability Through Phospholipid Complexation. Int. J. Mol. Sci..

[30] D’SOuza S.S., DeLuca P.P. (2006). Methods to assess in vitro drug release from injectable polymeric particulate systems. Pharm. Res..

[31] Zolnik B.S., Raton J.-L., Burgess D.J. (2005). Application of USP Apparatus 4 and In Situ Fiber Optic Analysis to Microsphere Release Testing. Dissolution Technol..

[32] Shen J., Burgess D.J. (2012). Accelerated in-vitro release testing methods for extended-release parenteral dosage forms. J. Pharm. Pharmacol..

[33] Nakmode D.D., Abdella S., Song Y., Garg S. (2025). Development of an in-situ forming implant system for levodopa and carbidopa for the treatment of parkinson’s disease. Drug Deliv. Transl. Res..

[34] (2009). Biological Evaluation of Medical Devices—Part 5: Tests for In Vitro Cytotoxicity.

[35] Uziel A., Gelfand A., Amsalem K., Berman P., Lewitus G.M., Meiri D., Lewitus D.Y. (2020). Full-Spectrum *Cannabis* Extract Microdepots Support Controlled Release of Multiple Phytocannabinoids for Extended Therapeutic Effect. ACS Appl. Mater. Interfaces.

[36] Costa M.S., Ramos A.M., Cardoso M.M. (2025). Drug Release Kinetics of PLGA-PEG Microspheres Encapsulating Aclacinomycin A: The Influence of PEG Content. Processes.

[37] Otte A., Park K. (2022). Transitioning from a lab-scale PLGA microparticle formulation to pilot-scale manufacturing. J. Control. Release.

[38] Son Y.J., Yun T.H., Lee J.G., Bang K.H., Kim K.S. (2024). Development and Characterization of Long-Acting Injectable Risperidone Microspheres Using Biodegradable Polymers: Formulation Optimization and Release Kinetics. Processes.

[39] Pathak P., Paliwal S. (2019). A Review on New Trends in Preparation of Long Acting Microspheres. J. Drug Deliv. Ther..

[40] Fan J.-B., Song Y., Wang S., Jiang L., Zhu M.-Q., Guo X. (2014). A synergy effect between the hydrophilic PEG and rapid solvent evaporation induced formation of tunable porous microspheres from a triblock copolymer. RSC Adv..

[41] Freiberg S., Zhu X.X. (2004). Polymer microspheres for controlled drug release. Int. J. Pharm..

[42] Bao Q., Wang X., Zou Y., Wang Y., Burgess D.J. (2022). In vitro release testing method development for long-acting injectable suspensions. Int. J. Pharm..

[43] Lozza I., Martín-Sabroso C., Hurtado-Marcos C., Montejo-Rubio C., Fraguas-Sánchez A.I., Torres-Suárez A.I. (2025). Cannabidiol-loaded-injectable depot formulation for the treatment of triple-negative breast cancer: Design, development, in-vitro and in-ovo evaluation of its anticancer activity. Int. J. Pharm..

[44] Kamali A., Oryan A., Hosseini S., Ghanian M.H., Alizadeh M., Eslaminejad M.B., Baharvand H. (2019). Cannabidiol-loaded microspheres incorporated into osteoconductive scaffold enhance mesenchymal stem cell recruitment and regeneration of critical-sized bone defects. Mater. Sci. Eng. C.

[45] Gómez-Lázaro L., Martín-Sabroso C., Aparicio-Blanco J., Torres-Suárez A.I. (2024). Assessment of In Vitro Release Testing Methods for Colloidal Drug Carriers: The Lack of Standardized Protocols. Pharmaceutics.

[46] Fraguas-Sánchez A.I., Fernández-Carballido A., Torres-Suárez A.I. (2021). Effect of Gamma Sterilization on CBD-Loaded PLGA Microparticles. Proceedings.

[47] Burgess D.J., Hussain A.S., Ingallinera T.S., Chen M.-L. (2002). Assuring quality and performance of sustained and controlled release parenterals: Workshop report. AAPS PharmSci.

[48] Massi P., Vaccani A., Bianchessi S., Costa B., Macchi P., Parolaro D. (2006). The non-psychoactive cannabidiol triggers caspase activation and oxidative stress in human glioma cells. Cell. Mol. Life Sci..

[49] Solinas M., Massi P., Cantelmo A., Cattaneo M., Cammarota R., Bartolini D., Cinquina V., Valenti M., Vicentini L., Noonan D. (2012). Cannabidiol inhibits angiogenesis by multiple mechanisms. Br. J. Pharmacol..

