# Supplementary material for: Development of Cannabidiol-Loaded PLGA Microspheres for Long-Acting Injectable Delivery: Evaluation of Poly(2-ethyl-2-oxazoline) as an Alternative to Poly(ethylene glycol)
Source: Pharmaceutics. 2026 Mar 8;18(3):336. doi: 10.3390/pharmaceutics18030336 (PMC13030583; doi:10.3390/pharmaceutics18030336)
Supplement: Supplementary file 1 [file pharmaceutics-18-00336-s001.zip › pharmaceutics-4161926-supplementary.pdf]

Supplementary material

Formulation Particle Size Analysis

Comparing formulations' (M1F, M2F, M4F, and M5F) particle size distribution using the Mastersizer.

	Record Number	Sample Name	Dx (10) (µm)	Dx (50) (µm)	Dx (90) (µm)
	1	M1F	26.9	62.1	125
	2	M1F	27.2	62.5	124
	3	M1F	27.3	62.1	122
Mean			27.2	62.2	124
1xStd Dev			0.187	0.224	1.47
1RSD (%)			0.69	0.361	1.19

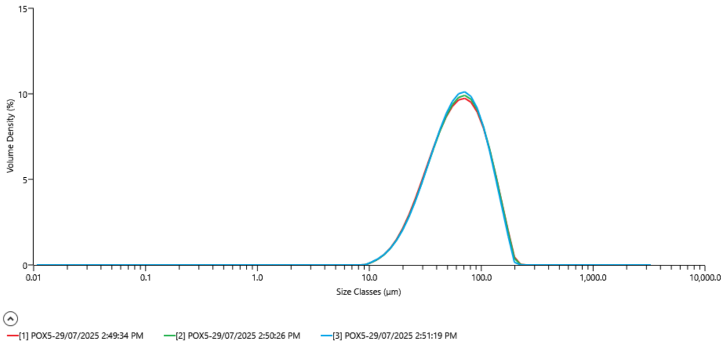

	Record Number	Sample Name	Dx (10) (µm)	Dx (50) (µm)	Dx (90) (µm)
	4	M4F	29.3	79.2	218
	5	M4F	29	79.1	204
	6	M4F	29	79.9	231
Mean			29.1	79.4	218
1xStd Dev			0.13	0.423	13.5
1RSD (%)			0.448	0.532	6.22

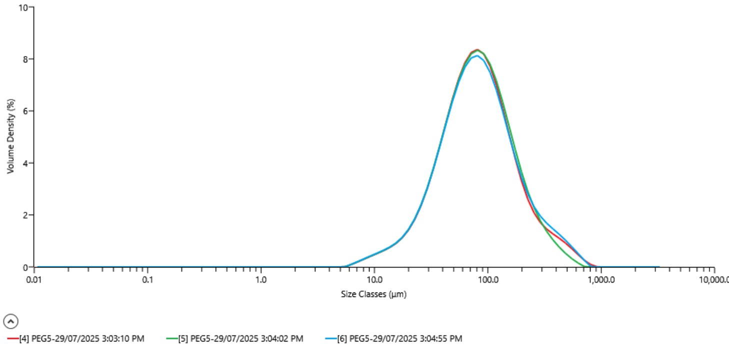

	Record Number	Sample Name	Dx (10) (µm)	Dx (50) (µm)	Dx (90) (µm)
	7	M2F	47.8	208	578
	8	M2F	47	210	570
	9	M2F	46.8	222	586
Mean			47.2	213	578
1xStd Dev			0.519	7.73	8.31
1RSD (%)			1.1	3.62	1.44

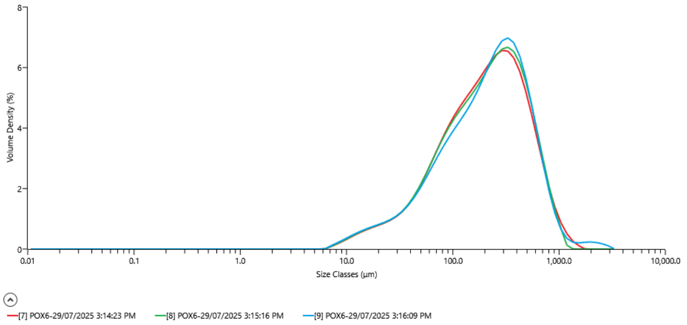

	Record Number	Sample Name	Dx (10) (µm)	Dx (50) (µm)	Dx (90) (µm)
	10	M5F	35.2	71.1	153
	11	M5F	34.2	68.1	133
	12	M5F	33.8	67.2	130
Mean			34.4	68.8	139
1xStd Dev			0.732	2.07	12.6

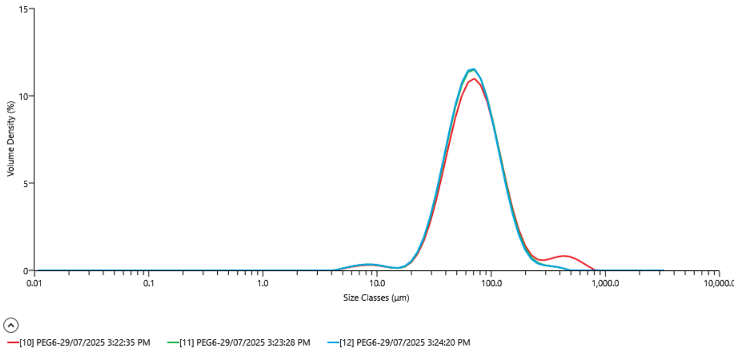

Additional SEM micrographs at various magnifications

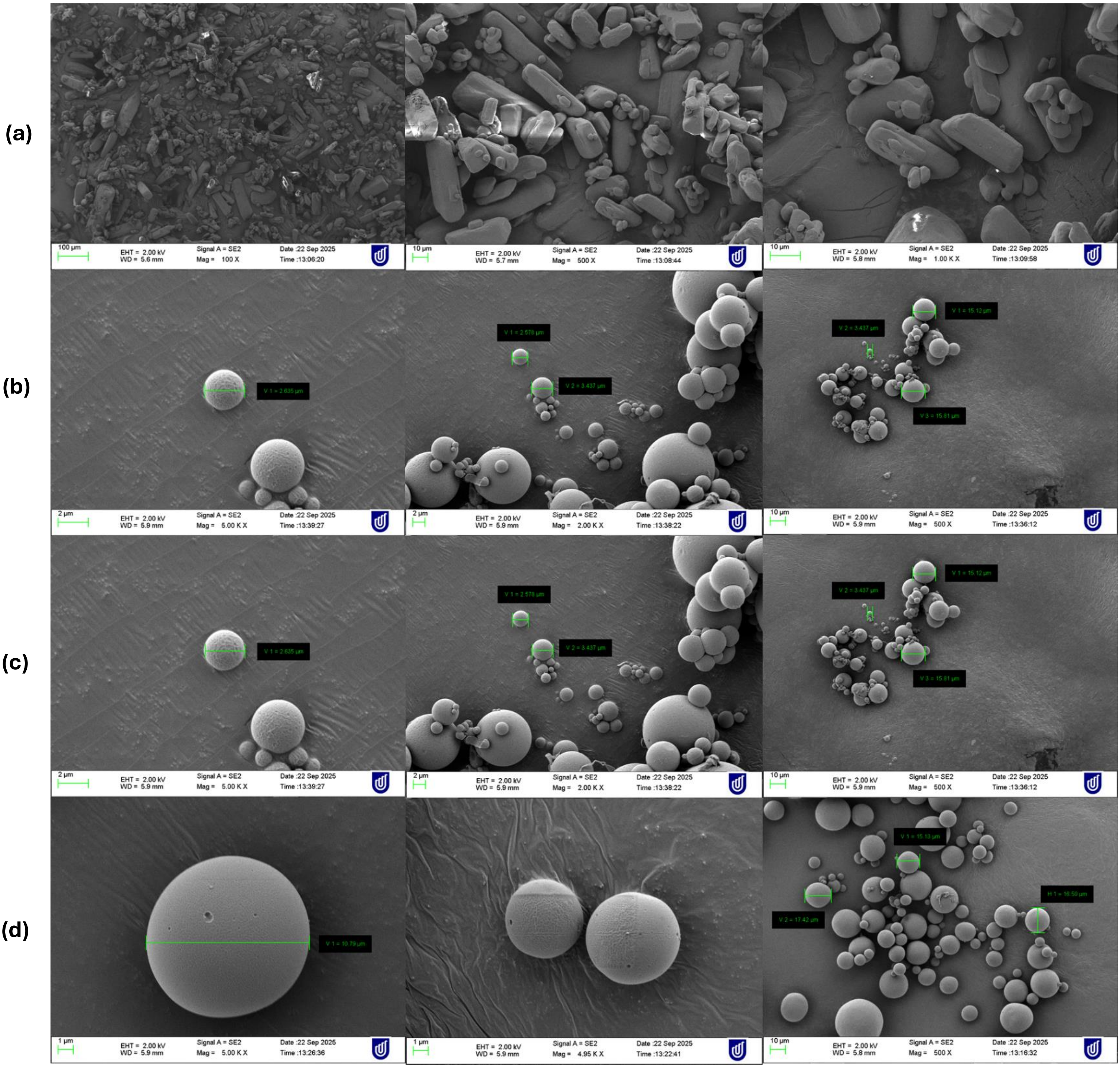

Images of cannabidiol and microspheres showing surface morphology examined using SEM. Here (a) is CBD only, (b) Formulation having no additive (M7F), (c) Formulation having PEG (M4F), and (d) formulation having POx (M1F).
